# Assessment of the relevance of midwifery competencies in academic education in Germany from the midwives’ perspective: A structural analysis of cross-sectional survey data

**DOI:** 10.18332/ejm/169658

**Published:** 2023-09-01

**Authors:** Angela Kranz, Anja A. Schulz, Markus A. Wirtz, Claudia Plappert, Harald Abele, Joachim Graf

**Affiliations:** 1 Section of Midwifery Science, Institute of Health Sciences, University of Tubingen, Tubingen, Germany; 2 Research Methods in the Health Sciences, University of Education Freiburg, Freiburg, Germany; 3 Department for Women’s Health, University Hospital Tubingen, Tubingen, Germany

**Keywords:** midwifery academization, academic midwifery competencies, survey instrument, psychometric testing, exploratory factor analysis, unpaired t-test

## Abstract

**INTRODUCTION:**

The acquisition of academic competencies is one of the main outcomes of the academization of midwifery education. To analyze midwives’ views on the key academic competencies of the recently reformed midwifery education in Germany, an existing assessment instrument was adapted to the German context of care and psychometrically analyzed. Furthermore, it was investigated whether the relevance assessments of academic and non-academic midwives differ from each other.

**METHODS:**

The study design was cross-sectional. A total of 193 (prospective) midwives answered the items on the assessed relevance of midwifery competencies in academic education (59 items); 3 items were added (referring to evidence-based practice and digital literacy). Construct validity was tested using exploratory factor analysis. Item and reliability analysis as well as unpaired t-tests were performed.

**RESULTS:**

Considering insufficient item-construct associations (20 items), a single factorial solution best fits the data (eigenvalue: 18.36; explained variance: 29.60%). Internal reliability was demonstrated to be very good with Cronbach’s α=0.954. The assessed relevance of academic midwifery competencies from academic and non-academic midwives did not differ significantly from each other for students and trainee midwives (t=0.18; df=6.66; p=0.86), and for for midwives educated at vocational school and university (t= -0.035; df=106; p=0.97).

**CONCLUSIONS:**

The adapted assessment tool can be used with minor modifications to reliably and validly measure the assessed relevance of academic competence from the midwives’ perspective. Combined with data on the assessments of medical practitioners and laypersons, the assessment provides a substantial data basis for the development of a competence profile for academic midwifery education in Germany.

## INTRODUCTION

Midwifery is continuously developing in Europe^1^. Social, health, and educational policies, as well as structural and demographic developments, have led to an expansion of the range of tasks and responsibilities of midwives. This has significant consequences for the acquisition of competencies in the context of professional education, especially focusing on academic competencies^1^. Generally, academic competencies are understood to be skills, attitudes, and behaviors that empower midwives to be able to meet the requirements in a complex care setting^2^. Accordingly, midwives should be particularly capable of self-responsibly adapting to new and changing challenges in an interdisciplinary care context, as well as making decisions and acting in a self-organized, participatory, and solution-oriented manner^3^. Thus, purposefully adopting evidence-based knowledge and interventions is essential^1^. These are developed through high-quality midwifery education, which can improve more than 50 outcomes, including reduced maternal and neonatal mortality and morbidity, a smaller number of unnecessary interventions, improved health and psychosocial outcomes, as well as lower rates of stillbirths and preterm births^4^.

### Structures of competence-oriented midwifery education in Germany

The need for midwives to acquire academic competencies led, among other things, to the academization of the midwifery profession and to the new midwifery law^1^. The academization is also progressing in Germany to meet the European standard of Directive 2005/36/EG^5^. Building on international^6^, European^7,8^ and national^3,9,10^ developments, it is therefore inevitable that academic education for midwives is competence-oriented. The new midwifery law (in force since 2020) has established the primary qualification at universities and colleges of academic education in Germany as the exclusive form of qualification for midwives^11^. The qualification to become a midwife in Germany takes place through a primary qualifying dual course of study. Academic education is organized according to the requirements of the Bologna Process^8^. This aims to achieve a standardized and integrative academic education in Europe, consisting of the three levels of bachelor’s, Master’s, and doctoral programs^8^. In Germany, the Bologna Process is implemented primarily through the German Qualifications Framework, which defines a consistent understanding of competencies for each qualification level^3^. In the midwifery study program, these academic competencies are taught in the 3 fields of action: pregnancy, birth and postnatal period, and breastfeeding^10^. After completing the study program, midwives receive occupational licensing to practice as a midwife as well as a Bachelor of Science degree. The study programs are offered either at medical faculties or at colleges in cooperation with healthcare institutions^1^. However, there is a transitional regulation for vocational training, in which vocational training at schools may be started by 2022 and must be completed by 2027^11^. Nevertheless, Germany has been offering primary academic qualifications since 2009, in addition to supplementary and integrated training courses^12^.

### Academic competence profiles in midwifery education

However, to examine the extent to which students possess academic competencies after completing their midwifery studies, it is a particular challenge to operationalize academic competencies as educational outcomes^1,6^. The International Confederation of Midwives focuses on basic competencies of midwifery practice that define core requirements for knowledge, skills, and professional behaviour^6^. Four superordinate categories are presented with all competencies considered essential: 1) General competencies; 2) Competencies specific to pre-pregnancy and antenatal care; 3) Competencies specific to care during labor and birth; and 4) Competencies specific to the ongoing care of women and newborns^6^. The World Health Organization recommends adopting these requirements nationally and consenting to these as outcomes of midwifery education^13^. Consequently, the operationalized competence profiles and the implemented curricula aligned to them vary systematically, especially between different countries.

The German Midwives Association addresses these basic competencies of the International Confederation of Midwives and adapts them to the specifics of German midwifery^9^. This results in five main categories: general competencies, specific competencies in the care of pregnancy, specific competencies during labor and birth, specific competencies during the postpartum and infant period, and specific competencies during pregnancy, birth, and postpartum^9^, which are further divided into individual competencies. These are explained by knowledge, skills, and personal competencies^9^. Another central competence profile for midwifery education originates in Switzerland^7^. Therefore, five domains: obstetric knowledge, obstetric communication, obstetric decision and action, responsibility and cooperation, development and organization^7^ are described with three to six competencies, each corresponding to the described field of action^7^. In this context, Pehlke-Milde^7^ has developed an instrument for the assessment of the views of midwives on competence requirements. Several items ask about the mentioned domains.

In Germany, there is so far no consented and validated instrument that adequately captures the achievement of the competence goals of academic midwifery education. In order to be able to specify the assessment contents appropriately, the perspective of midwives is a relevant source of information in addition to conceptual specifications for the qualification of midwives.

To achieve this particular aim, in a first step, the existing survey instrument of Pehlke-Milde^7^ assessing individual views on academic midwifery competencies will be adapted to the German midwifery context and psychometrically tested. Secondly, in order to comprehensively represent the views of midwives, both professional and academic midwives (in education and licensed midwives) will be surveyed.

Two main research questions are addressed in this study: ‘Is the adopted instrument a reliable and valid tool to measure the assessed relevance of academic midwifery competencies from the midwives’ perspective?’, and ‘Do academic and non-academic midwives differ significantly from each other in their assessed relevance of academic midwifery competencies?’.

## METHODS

### Design

The one-time cross-sectional survey was conducted as part of the research project ‘Good Midwife’ at the University of Tübingen. The aim is to develop an empirically tested competency model for academic midwifery education in Germany. In a first step, the assessed relevance of midwives, health professionals, and laypersons on academic key competencies in midwifery education is collected and analyzed. In a future research phase, the expectations of pregnant women about midwives’ competencies will be considered, for which further data will be collected with the present questionnaire. The focus of this work is on the perspective of (prospective) midwives.

### Recruitment and study sample

Recruitment of midwives and prospective midwives took place from April to October 2022. Prospective midwives (students or trainees) and licensed midwives (academic or non-academic) were included if they: 1) were of legal age, 2) had sufficient knowledge of German, and 3) gave informed consent. Recruitment took place in three stages.

*Midwifery students*

An email invitation was sent to all midwifery students registered at University of Tübingen at the time of recruitment. Additionally, several midwifery programs across Germany (n=5) were contacted and asked to send the study invitation to midwifery students.

*Midwifery trainees*

The last regular cohorts of midwifery trainees at University Hospital Tübingen were also contacted by e-mail for participation in the study.

*Licensed midwives*

Invitations to participate in the study were sent to freelance midwives (n=25) and various clinics with midwifery staff (n=5).

In addition, multipliers were used to increase the recruitment radius: calls for studies were placed on the websites of midwife-specific professional associations (Regional Association of Midwives in Baden-Württemberg, Society for Quality in Outpatient Obstetrics) and in professional journals (e.g. Hebamme, publisher: Thieme).

Participants were informed about the purpose of the study, voluntary participation, anonymous data collection, data storage and use, and participants’ rights. Data collection, storage, and analysis were carried out in accordance with the General Data Protection Regulation^14^. Out of 307 questionnaires started, 193 were completed (of which all could be evaluated). At this point, it is interesting to take a look at the number of practicing midwives in Germany. Approximately 27000 midwives are practicing in Germany (as of 2021)^15^. With n=108 licensed midwives in the present study (excluding midwifery students and midwifery trainees), this results in a rate of approximately 0.4% of the midwives in Germany who participated in the study. In terms of the sample size for psychometric testing, the recommendation of n>100 was considered^16^.

### Assessment instrument

In order to capture the midwives’ view, the Pehlke-Milde^7^ instrument for the assessment of the competence requirements of midwives was adapted to the context of midwifery in Germany. The instrument contains a total of 59 items divided into 7 thematic areas: effective and efficient care (16 items), legal and ethical responsibilities (5 items), interdisciplinary care (3 items), comprehensive care and prevention (10 items), professional relationships (8 items), analysis and integration of knowledge (10 items), and expending and deepening of professional knowledge (7 items). In the present work, three items VI (midwife’s research skills), VI5 (digital competences of the midwife), and II6 (avoiding avoidable interventions) were newly added. These addressed current challenges in midwifery and focused on evidence-based practice and digital literacy^9^. In addition, all items were rearranged. The new arrangement of the items was based on eight domains based on the competency profile of the International Confederation of Midwives^6^ as well as the German Midwives Association^9^ to reflect current developments. In total, the adapted assessment consisted of 62 items to assess the perceived relevance of midwifery competencies in academic education in Germany from the perspective of midwives. The questionnaire consisted of statements (e.g. ‘The midwife meets legal requirements and supports the development of evidence-based standards’) for which the respondents could rate the relevance of the statement on a 6-point scale (1=highest priority to 6=no priority). Expert interviews were used to test the content validity of the instrument. An analysis of the construct validity and, thus, the empirical identification of the competence structure is not available. At the item level, the instrument mainly reflects the range of content areas and academic competencies according to the curricular design of German midwifery education and addresses core competencies according to the International Confederation of Midwives^6^.

In addition, 12 questions were asked about the sociodemographic characteristics of the participants (based on the German Demographic Standard^17^). The final instrument was pre-tested using cognitive interviews^18^, resulting in minor changes. The final data collection was carried out using the online survey software LimeSurvey.

### Statistical analysis

To test for normal distribution, the skewness (S) and kurtosis values were calculated for each item. The cut-off level for a normal distribution was set at |S|<3.00 and |kurtosis|<7.00^19^. Unpaired t-tests were used to capture the differences in means to identify possible differences in the assessed relevance of academic midwifery competencies between the two groups of: 1) academic vs non-academic midwives, and 2) midwife trainees vs midwife students. Cohen’s d was used as a measurement of the effect size^20^. Subdivisions of d=|0.20| (small effect), d=|0.50| (medium effect) and d=|0.80| (large effect) were defined^20^. The alpha level for all statistical tests was set at 0.05.

Due to the lack of empirical testing of the construct validity of the Pehlke-Milde^7^ instrument, no empirical evidence exists regarding the multidimensionality of the assessment. The heuristic procedure of exploratory factor analysis provided hypotheses about the structure of the relationships between the items. This procedure was useful when there is no elaborated theory and the linear structures of the measured characteristics are to be explored^21^, as in the present study. Using principal components analysis with varimax rotation and exploratory factor analysis, the 62 items were analyzed. The communalities (h^2^i) were calculated, which were accepted from a cut-off level h^2^i>0.40^22^. To identify relevant factors, the Kaiser-Guttman criterion (eigenvalue >1.00) and the scree plot criterion were conducted. Furthermore, an item was assigned to a factor if the rotated factor loading (λ') was >0.50^22^. To calculate the internal reliability, the reliability coefficient Cronbach’s α was used^23^. It determined the internal consistency of a scale^23^. In psychometric procedures, satisfactory reliability was achieved by the following cut-off levels: α≥0.70 acceptable, α≥0.80 good, and α≥0.90 very good^22^. Values of α ≤0.50 were not acceptable^23^. Furthermore, the corrected item discriminatory power (r_it_) was calculated. Data analysis was carried out using the statistical software Statistical Package for the Social Sciences (SPSS) version 26.

## RESULTS

### Participant characteristics

Table 1 shows the sociodemographic characteristics of study participants. A total of n=193 people participated in the survey and 99.5% (n=192) of the participants were female, 29.5% (n=57) of participants were aged 21–29 years, while 20.2% (n=39) were aged 50–59 years; 40.4% (n=78) of the participants reported completing vocational school as their highest professional education degree. In contrast, 17.6% (n=34) had a Bachelor’s degree, 9.8% (n=19) a Master’s degree or comparable, and 4.1% (n=8) a diploma; 38.3% of participants (n=74) were employed full-time, while 20.7% (n=40) were employed part-time, and 26.9% (n=52) were students. Almost half of the participants reported being a midwife educated in a vocational school (46.6%, n=90), and 9.3% (n=18) were midwives educated at university; 3.6% (n=7) of participants were midwifery trainees, whereas 28.5% (n=55) were midwifery students. In addition, n=2 physicians (1.0%) and n=3 other medical personnel (1.6%) participated. Overall, 90.7% of participants were medical personnel (n=175), whereas 12.4% (n=24) were laypersons, and 64.2% (n=124) of the respondents belonged to a religion.

**Table 1:** Sociodemographic characteristics of study participants (N=193)

*Characteristics*	*n (%)*
**Gender**	
Female	192 (99.5)
Male	1 (0.5)
Other	0 (0.0)
**Age** (years)	
18–20	12 (6.2)
21–29	57 (29.5)
30–39	42 (21.8)
40–49	33 (17.1)
50–59	39 (20.2)
60–69	10 (5.2)
≥70	0 (0.0)
**Marital status**	
Married	98 (50.8)
Registered partnership	1 (0.5)
Divorced	9 (4.7)
Widowed	1 (0.5)
Single	84 (43.5)
**Living with partner in household**	
Yes	127 (65.8)
No	66 (34.2)
**Pregnancy**	
Yes	8 (4.1)
No	185 (95.9)
**Number of children**	
1	25 (13.0)
2	42 (21.8)
3	28 (14.5)
≥4	13 (6.7)
0	85 (44.0)
**Highest general school degree**	
No degree	0 (0.0)
Secondary school diploma	22 (11.4)
Polytechnic secondary school in the GDR with completion of the 8th or 9th grade	0 (0.0)
Intermediate school	0 (0.0)
Polytechnic secondary school in the GDR with completion of 10th grade	2 (1.0)
Advanced technical college	14 (7.3)
‘Abitur’/general or subject-linked higher education entrance qualification	154 (79.8)
Other general school degree	1 (0.5)
**Highest vocational qualification**	
No vocational qualification	9 (4.7)
Still in vocational training	35 (18.1)
Completed vocational training	78 (40.4)
Completion of a master craftsman or technician school	1 (0.5)
Bachelor’s degree	34 (17.6)
Diploma	8 (4.1)
Master’s, Magister, state examination	19 (9.8)
Doctorate	5 (2.6)
Habilitation	1 (1.0)
Other professional degree	3 (1.6)
**Parents’ highest vocational qualification**	
No vocational qualification	4 (2.1)
Still in vocational training	0 (0.0)
Completed vocational training	77 (39.9)
Completion of a master craftsman or technician school	20 (10.4)
Bachelor’s degree	7 (3.6)
Diploma	40 (20.7)
Master’s, Magister, state examination	27 (14.0)
Doctorate	11 (5.7)
Habilitation	3 (1.6)
Other professional degree	4 (2.1)
**Employment situation**	
Full-time employed	74 (38.3)
Part-time employed	40 (20.7)
Partial retirement	0 (0.0)
Marginally employed	2 (1.0)
‘One-Euro-Job’ (when receiving unemployment benefit II)	0 (0.0)
Other occupation	0 (0.0)
Occasionally or irregularly employed	1 (0,5)
In vocational training/apprenticeship with earnings	9 (4.7)
In retraining	0 (0.0)
Voluntary military service	0 (0.0)
Federal voluntary service or voluntary social year	1 (0.5)
Maternity leave, parental leave or other leave of absence	11 (5.7)
Pupils at a general education school	0 (0.0)
Students	52 (26.9)
Pensioners, retirees, early retirees	2 (1.0)
Unemployed	0 (0.0)
Permanently disabled	0 (0.0)
Housewives/househusbands	0 (0.0)
Other	1 (1.0)
**Occupation of healthcare professionals (N=175)**	
Midwifery trainees	7 (3.6)
Midwifery students	55 (28.5)
Midwife (vocational school educated)	90 (46.6)
Midwife (university educated)	18 (9.3)
Physician	2 (1.0)
Psychologist	0 (0.0)
Other healthcare professionals	3 (1.6)
**Occupation of laypersons (N=24)**	
Self-employed in trade, hospitality, crafts, industry, services	4 (2.1)
Civil servant, judge, professional soldier	4 (2.1)
Employee with executive activity according to general instructions	1 (0.5)
Employee with a qualified activity that is performed according to instructions	5 (2.6)
Employee with independent performance in a responsible position or with specialist responsibility for personnel	3 (1.6)
Employee with comprehensive management duties and decision-making powers	1 (0.5)
Worker	2 (1.0)
In vocational training/apprenticeship	3 (1.6)
Other profession	1 (0.5)
**Religious affiliation**	
Yes	124 (64.2)
No	69 (35.8)

### Exploratory factor analysis

**Figure 1:**
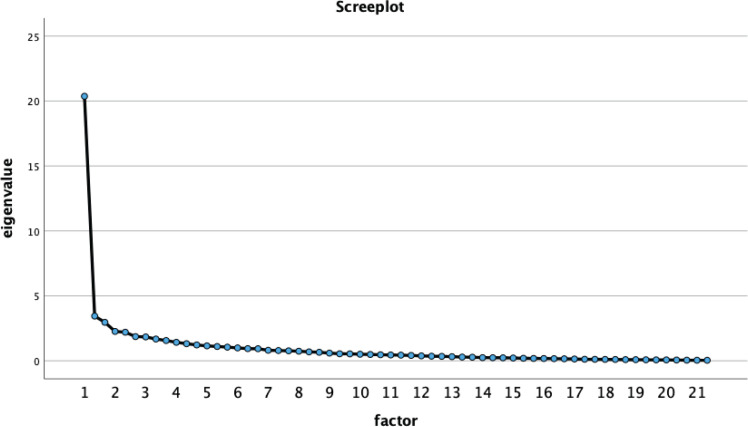
Screeplot. Output SPSS.

The commonalities of the items using the extraction method of principal components analysis are shown in Table 2. All items were above the cut-off level h^2^i >0.40, meaning that the proportion of the total variance of a variable that can be attributed to the common factors was acceptable for all variables. Table 3 shows the factor eigenvalues and the explained total variance. According to the Kaiser-Guttman criterion (eigenvalue >1.00), 15 factors were extracted. These 15 factors explain 66.27% of the original information held by all items. It should be noted that factor one explains 29.60% (eigenvalue: 18.36). The scree plot assumed a one-factorial solution (Figure 1). Table 2 shows the item loadings on the extracted factors from the rotated component matrix, and 42 items exceeded the cut-off level λ' >0.50. All these items loaded on one single factor, 20 items did not exceed the cut-off-level λ' >0.50. These items, therefore, were eliminated. After elimination, all items exhibited factor loadings >0.50 (except item III5, burn-out prophylaxis) and possessed a common source of variance (latent factor 1). The eigenvalues and the scree plot did not change remarkably. Based on the results of the exploratory factor analysis, the 42 items were confirmed as indicators of an underlying latent construct (relevance of academic midwifery competencies, unidimensional structure).

**Table 2:** Item loadings on extracted factors and communalities from rotated component matrix

*Item*	λ' ^a^*	λ' ^a^**	*h^2^i ^b^*
II1 The midwife’s ability to make decisions and take action	0.293	-	0.731
II2 Role of the midwife as primary caregiver and supporter	0.374	-	0.658
II3 Maintenance and promotion of women’s health	0.421	-	0.685
II4 Safety, effectiveness and efficiency of care	**0.523**	**0.506**	0.751
II5 Identification of care needs and care in crises	**0.564**	**0.562**	0.592
II6 Avoiding avoidable interventions	**0.533**	**0.525**	0.634
III1 Broadening and deepening theoretical and scientific knowledge	0.395	-	0.726
III2 Planning and reflection in terms of safe, effective and efficient care	**0.528**	**0.538**	0.606
III3 Identify factors influencing women’s and children’s health	**0.626**	**0.629**	0.648
III4 Understanding legal and ethical principles	**0.716**	**0.721**	0.680
III5 Burn out prophylaxis	**0.500**	0.495	0.679
IV1 Compliance with legal requirements and support for evidence-based practice	**0.533**	**0.546**	0.597
IV2 Physiological support of the process and evidence-based action	0.447	-	0.647
IV3 Assessment and response to obstetric risks	0.463	-	0.613
IV4 Midwife’s internal evidence	0.497	-	0.663
V1 Midwife’s research skills	**0.522**	**0.547**	0.738
V2 Evaluate and derive evidence from scientific information	**0.559**	**0.589**	0.753
V3 Knowledge of women’s health	**0.649**	**0.671**	0.690
V4 Traditional and experiential knowledge	**0.561**	**0.580**	0.583
V5 Coping with job-specific demands	**0.646**	**0.652**	0.696
V6 Representing opinions in hierarchical structures	**0.641**	**0.658**	0.669
V7 High level of professional performance through continuing education	**0.652**	**0.661**	0.658
VI1 Constant expansion of knowledge	**0.577**	**0.592**	0.754
VI2 Gathering information and analyzing the situation	**0.617**	**0.625**	0.667
VI3 Sensory perception (tactile-kinesthetic, body-therapeutic)	**0.518**	**0.501**	0.707
VI4 Integration of learning processes into professional action	**0.644**	**0.658**	0.611
VI5 Digital competences of the midwife	**0.511**	**0.532**	0.607
VII1 Dignity and respect in the relationship of trust between woman and midwife	0.446	-	0.704
VII2 Respect for individual, social, cultural, religious and emotional needs of the woman	0.436	-	0.655
VII3 Professional relationship building	**0.538**	**0.524**	0.648
VII4 Reflecting asymmetrical power relations	0.468	-	0.660
VII5 Professional role relationship	**0.637**	**0.637**	0.662
VII6 Therapeutic working alliance with the woman	**0.602**	**0.588**	0.722
VII7 Respect for the woman’s own competence and autonomy	0.480	-	0.699
VII8 Respecting and promoting pregnancy, birth, puerperium and breastfeeding as life events of the woman	0.453	-	0.722
VII9 Recognizing mother and child as a unit, including mother and family	0.472	-	0.686
VII10 Resource-oriented inclusion of the woman’s family environment	**0.625**	**0.617**	0.651
VII11 Women- and family-oriented care	**0.636**	**0.631**	0.700
VII12 Education and counselling of women and families	0.452	-	0.649
VII13 Educating adolescents about sexuality and pregnancy	0.451	-	0.693
VII14 Recognizing and referring signs of violence, sexual abuse or drugs	**0.639**	**0.608**	0.722
VII15 Identify deficits in care and child abuse and act	**0.535**	**0.504**	0.693
VIII1 Decide within legal competence and involve other professionals	**0.519**	**0.512**	0.657
VIII2 Involve physicians in cases with pathological findings	0.327	-	0.666
VIII3 Ensure integrated care	**0.606**	**0.608**	0.605
VIII4 Meeting multiple demands and setting priorities	**0.584**	**0.588**	0.462
VIII5 Assume responsibility in the obstetric team	0.493	-	0.692
VIII6 Promote optimal interdisciplinary cooperation	**0.605**	**0.617**	0.685
VIII7 Adequate documentation and dissemination of information to lay and professional representatives	**0.552**	**0.584**	0.618
IX1 Understand obstetric care, considering relevant theories, principles and methods	**0.597**	**0.610**	0.676
IX2 Advocacy for national and international social and health policies	**0.597**	**0.619**	0.699
IX3 Reference to national and international standards and codes of ethics	**0.652**	**0.674**	0.682
IX4 Identification of ethical dilemmas and participation in ethical decision-making processes	**0.641**	**0.664**	0.683
IX5 Exercising professional responsibility and liability	**0.588**	**0.583**	0.544
IX6 Realize working conditions for safe, effective and efficient care	**0.652**	**0.650**	0.591
IX7 Promoting the reputation of the profession	**0.536**	**0.541**	0.691
IX8 Recognize and advocate for the social relevance of professional performance	**0.624**	**0.634**	0.623
IX9 Guidance and counselling for new entrants to the profession	0.466	-	0.616
IX10 Observe legal, economic and business principles	0.414	-	0.652
IX11 Internal and external evaluation of performance	0.401	-	0.623
IX12 Support health promotion and prevention	**0.536**	**0.552**	0.666
IX13 Autonomy and responsibility according to ethical, legal and scientific principles	**0.507**	**0.511**	0.647

a λ': rotated factor loading.

* Rotated factor loading for all items.

** Rotated factor loading after elimination of 20 items.

b h^2^i: communalities.

Bold print means factor loading λ' >0.50.

**Table 3:** Eigenvalue and explained variance by extraction method principal component analysis

*Component*	*Eigenvalue*	*% of explained variance*	*Cumulated %*
1	18.356	29.606	29.606
2	3.035	4.896	34.502
3	2.686	4.333	38.835
4	1.944	3.135	41.971
5	1.786	2.881	44.852
6	1.684	2.716	47.567
7	1.610	2.597	50.164
8	1.588	2.562	52.726
9	1.373	2.214	54.940
10	1.303	2.102	57.042
11	1.274	2.054	59.096
12	1.237	1.995	61.091
13	1.105	1.782	62.873
14	1.062	1.713	64.585
15	1.046	1.688	66.273

### Internal consistency and item analysis

Table 4 lists the descriptive analysis of the items, the item difficulty, the item total correlations, and the test of deviations from the normal distribution. The reliability analysis was conducted for all 42 items of the one factor, with Cronbach’s α=0.954. Accordingly, the reliability of the overall scale and the internal consistency were very good. The adapted assessment reliably captures the assessed relevance of the academic competencies of midwives. The item discriminatory power (considering all items) showed several weaknesses (r_it_ <0.40, Table 4) for some items that also showed weaknesses in factor loadings (λ' <0.50). After the elimination of several items as described previously, the item discriminatory power was above the cut-off level r_it_ >0.40 for all considered items. This meant that these items predicted acceptable item differentiation.

**Table 4:** Descriptive analysis of the items, item difficulty, test for normal distribution and item discriminatory power (N=193)

*Item*	*Mean^a^*	*SD*	*Range*	*Mode*	*Median*	*S^b^*	*Kurtosis^c^*	*r_it_^d^*	*r_it_^e^*
II1	1.41	0.589	1–4	1	1	1.438	2.615	0.278	-
II2	1.31	0.537	1–3	1	1	1.516	1.393	0.350	-
II3	1.34	0.566	1–3	1	1	1.433	1.093	0.394	-
II4	1.61	0.629	1–3	1	2	0.541	-0.617	0.491	0.473
II5	1.75	0.679	1–4	2	2	0.464	-0.370	0.536	0.532
II6	1.44	0.610	1–4	1	1	1.364	2.134	0.503	0.494
III1	1.77	0.639	1–4	2	1	0.037	-0.092	0.037	-
III2	1.73	0.707	1–4	2	2	0.705	0.268	0.504	0.511
III3	1.89	0.755	1–4	2	2	0.412	-0.489	0.594	0.593
III4	2.21	0.772	1–4	2	2	0.095	-0.491	0.691	0.692
III5	2.10	0.797	1–4	2	2	0.311	-0.386	0.472	0.460
IV1	2.19	0.823	1–4	1	2	0.084	-0.732	0.518	0.521
IV2	1.54	0.684	1–4	1	1	1.089	0.721	0.418	-
IV3	1.31	0.545	1–4	1	1	1.799	3.413	0.438	-
IV4	1.69	0.718	1–4	1	2	0.701	-0.133	0.462	-
V1	3.01	0.971	1–5	3	3	-0.079	-0.355	0.513	0.053
V2	2.53	0.907	1–5	2	3	0.089	-0.602	0.545	0.567
V3	2.07	0.791	1–5	2	2	0.454	0.223	0.629	0.645
V4	2.05	0.870	1–5	2	2	0.569	-0.044	0.536	0.551
V5	2.33	0.862	1–5	2	2	0.237	-0.316	0.630	0.625
V6	1.85	0.743	1–4	2	2	0.471	-0.332	0.616	0.630
V7	2.38	0.865	1–5	3	2	0.097	-0.177	0.626	0.628
VI1	2.09	0.798	1–5	2	2	0.389	0.071	0.563	0.569
VI2	2.07	0.872	1–5	2	2	0.487	-0.204	0.590	0.598
VI3	1.93	0.813	1–5	2	2	0.780	0.676	0.483	0.466
VI4	2.19	0.754	1–4	2	22	0.191	-0.305	0.626	0.634
VI5	2.91	0.972	1–6	3	3	0.212	0.029	0.498	0.507
VII1	1.33	0.561	1–4	1	1	1.702	2.897	0.421	-
VII2	1.40	0.623	1–4	1	1	1.415	1.483	0.414	-
VII3	1.67	0.694	1–4	1	2	0.728	0.107	0.519	0.500
VII4	2.02	0.869	1–5	2	2	0.547	-0.138	0.448	-
VII5	2.20	0.843	1–5	2	2	0.455	0.259	0.621	0.613
VII6	2.25	0.964	1–6	2	2	0.703	0.602	0.580	0.560
VII7	1.47	0.669	1–4	1	1	1.335	1.361	0.451	-
VII8	1.31	0.545	1–3	1	1	1.604	1.663	0.423	-
VII9	1.40	0.605	1–3	1	1	1.251	0.522	0.442	-
VII10	1.87	0.723	1–4	2	2	0.293	-0.765	0.596	0.583
VII11	1.90	0.781	1–4	2	2	0.439	-0.509	0.604	0.596
VII12	1.46	0.629	1–4	1	1	1.185	0.911	0.428	-
VII13	2.83	1.112	1–6	3	3	0.389	0.014	0.432	-
VII14	1.82	0.771	1–4	2	2	0.659	-0.015	0.618	0.578
VII15	1.52	0.654	1–3	1	1	0.891	-0.306	0.509	0.474
VIII1	1.59	0.624	1–3	1	2	0.563	-0.597	0.490	0.478
VIII2	1.26	0.508	1–3	1	1	1.775	2.333	0.306	-
VIII3	1.84	0.741	1–4	2	2	0.646	0.266	0.573	0.573
VIII4	1.77	0.792	1–4	1	2	0.823	0.167	0.558	0.555
VIII5	2.12	0.869	1–6	2	2	1.006	2.831	0.469	-
VIII6	2.13	0.809	1–4	2	2	0.176	-0.654	0.581	0.588
VIII7	1.90	0.777	1–4	2	2	0.653	0.176	0.534	0.563
IX1	2.31	0.927	1–6	2	2	0.583	0.745	0.577	0.584
IX2	2.60	1.081	1–6	3	3	0.324	-0.102	0.583	0.598
IX3	2.56	0.972	1–6	3	3	0.177	-0.168	0.639	0.655
IX4	2.69	1.023	1–6	3	3	0.201	-0.112	0.631	0.646
IX5	2.21	1.032	1–6	2	2	0.628	0.190	0.567	0.556
IX6	2.33	0.914	1–5	2	2	0.215	-0.373	0.639	0.628
IX7	2.42	0.899	1–5	3	2	0.141	-0.327	0.524	0.521
IX8	2.40	0.909	1–5	3	2	0.120	-0.382	0.608	0.610
IX9	2.05	0.858	1–5	2	2	0.549	-0.019	0.457	-
IX10	2.21	0.843	1–6	2	2	0.847	1.580	0.398	-
IX11	2.64	1.119	1–6	2	3	0.529	0.119	0.396	-
IX12	2.66	0.944	1–6	3	3	0.421	0.340	0.528	0.533
IX13	2.05	0.917	1–6	2	2	0.757	0.997	0.489	0.488

a Arithmetic mean: 1=highest priority; 2=high priority; 3=rather high priority; 4=rather low priority; 5=low priority; and 6=no priority. SD: standard deviation.

b S: skewness; standard error of skewness = 0.175.

c Standard error of kurtosis = 0.348.

d r_it_: corrected item discriminatory power acceptable values from r_it_ >0.40 for all items.

e r_it_ : corrected item discriminatory power acceptable values from r_it_ >0.40 after eliminated items.

The arithmetic means differed in the value ranging between 1.26 (item VIII2 – involve physicians within pathological findings) and 3.01 (item V – midwives’ research skills), which also represented the item difficulties. According to the Classical Test Theory, items with an arithmetic mean close to 3.00 are preferred for a 6-point scale^24^. This was captured within the items V1 (midwife’s research skills), V2 (evaluate and derive evidence from scientific information), VI5 (digital competencies of the midwife), VII13 (educating adolescents about sexuality and pregnancy), IX2 (advocacy for national and international social and health policies), IX3 (reference to national and international standards and codes of ethics), IX4 (identification of ethical dilemmas and participation in ethical decision-making processes), IX11 (internal and external evaluation of performance), and IX12 (support health promotion and prevention). These items had a higher informational content than the other items. For the other items, the item difficulty had a mean <2.50. A floor effect of the item difficulty was therefore determined. The skewness and kurtosis values were |S|<3.00 and |kurtosis|<7.00, which means that a standard distribution was assumed.

### Differences in the assessed relevance of academic midwifery competencies

Table 5 shows the unpaired t-test for students and trainees of midwifery. There was no significant difference between students and trainees in midwifery (t=0.18; df=6.66, p=0.86). The students and trainee midwives do not differ significantly in their assessed relevance of academic midwifery competencies. The unpaired t-test for midwives educated at university and those educated at vocational school is shown in Table 5. There was no significant difference in the assessed relevance of academic midwifery competencies between the midwives educated at university and midwives educated at vocational school (t= -0.035; df=106, p=0.97).

**Table 5:** Unpaired t-test for student or trainee midwives, and for midwives educated at university or at vocational school

*Midwives*				*Levene test*		*Unpaired t-test*		
	*n*	*Mean^a^*	*SD*	*F*	*sig.^b^*	*t*	*df*	*sig.^b^*
Students	55	2.062	0.446	5.43	0.023	0.183	6.657	0.861
Trainees	7	2.013	0.687	5.43	0.023	0.183	6.657	0.861
University educated	18	2.103	0.500	0.75	0.388	-0.035	106	0.972
Vocational school educated	90	2.107	0.504	0.75	0.388	-0.035	106	0.972

a Scale arithmetic mean. SD: standard deviation.

b sig.: significance according to alpha level of 5% (α≤0.05).

## DISCUSSION

This study tested the questionnaire structure as well as differences in the views of academic and non-academic midwives in relation to the assessed relevance of academic midwifery competencies. A total of n=193 respondents participated in the survey. A single factorial solution was extracted after eliminating 20 items due to a lack of quality. The internal reliability of the assessment was very good (α=0.954). Accordingly, the first research question can be answered as follows: The assessment instrument measured the relevance of academic midwifery competencies reliably as well as construct validity. Focusing on the characteristics of academic midwifery competencies, the assessed relevance for academic midwives and non-academic midwives did not differ significantly from each other (t=0.18; df=6.66, p=0.86 for students and trainee midwives; and t= -0.035; df=106, p=0.97 for midwives educated at vocational school and university) answering the second research question (‘Do academic and non-academic midwives differ significantly from each other in their assessed relevance of academic midwifery competencies?’).

There was consensus among the midwives’ relevance of academic midwifery competence, as the performed t-tests both demonstrated no significant difference in the assessed relevance of academic midwifery competencies of the academic and non-academic midwives. This consensus about the academization of the profession was already reflected in other studies^25^. Further research shows that all stakeholders in academization, including non-academic midwives, unanimously supported the academization of midwifery^26^. However, fundamental differences were also found in the assessments of the two groups of academic and non-academic midwives in terms of shaping the academization^25-28^. Accordingly, it can be concluded that there is consensus among the midwifery profession about the need for academic midwifery competence, which can be supported by the results of the non-significant t-tests as well as the one factorial solution presented here. However, it should be noted that the views in terms of shaping the academization of midwifery differ between academic and non-academic midwives according to the current state of research, as described before. The fact that no studies were identified that found non-significant differences in the perceptions of academic and non-academic midwives could be due to publication bias. This bias assumed that studies that did not find statistically significant differences were published less frequently or later than studies with significant results^29^.

Considering the descriptive analysis, it appears that on average, most of the items were rated as ‘highest priority’ and ‘high priority’. This showed that there was fundamental agreement among the respondents about the characteristics of competent academic midwifery. However, one item (V1 – research competency) had a mean >3.00 and was therefore rated as ‘rather high priority’. This is particularly concerning since academization pursued the goal of evidence-based practice^30^. Considering the sociodemographic characteristics of the respondents, it should be noted that the majority of the midwives participating were non-academic midwives (46.6%). This indicated that there was a need for education to sensitize and involve non-academic midwives in terms of the goals of academization. Indeed, even for midwives who were already licensed, it was central to engage with research skills and evidence to achieve quality healthcare^31^. The need for academically trained midwives has been nationally and internationally undisputed for decades^26^. Accordingly, this single-factor solution that was extracted is highly relevant in terms of content since the construct reflected the relevance assessment of midwifery competency based on the academization of the midwifery profession from the midwives’ perspective.

However, it is debatable to what extent the items represented the construct fully. Items that were eliminated due to low item quality showed relevant content. For example, items relating to the interdisciplinarity of the midwifery profession (item VIII2 – involve physicians in cases with pathological findings) or to the expansion of scientific expertise (item III1 – broadening and deepening theoretical and scientific knowledge) had to be excluded, although their content was not included in other items that were part of the factor solution. It was also possible that the extracted items belonged to other factors that are not represented in the data set. They may nevertheless be relevant to the construct as a whole and just not adequately represented in the present questionnaire. Due to the one-factorial solution, a very large amount of information has been lost (one factor explained 29.6% of the overall variance, leaving almost 70% of the variance unexplained).

Considering the reliability hypothesis (H1: The measurement instrument can reliably capture the assessed relevance of competence facets of midwives), it must be mentioned that the prerequisites for Cronbach’s α were not completely fulfilled. Although one-dimensionality was given, the covariances of the items were not identical. Accordingly, the assessment of reliability could be biased^24^. Nevertheless, Cronbach’s α was applied because it was the best known and most frequently used reliability coefficient^24^. Finally, the quality criteria of objectivity and reliability were fulfilled. Validity was given in terms of construct validity (through exploratory factor analysis) as well as content validity (through expert interviews by Pehlke-Milde^7^). The criterion of validity, however, was not fulfilled.

### Strengths and limitations

The study, has some strengths, such as use of the methodology of Classical Test Theory that has been proven and tested many times^32^, and the prerequisites of the statistical procedures were met in most cases (e.g. normal distribution). Also, the hypotheses could be fully tested, and research questions could be fully answered. Nevertheless, the study has some limitations. It should be noted that the distinction used between academic and non-academic midwives refers to the primary education of midwives. A secondary academization of the midwife (e.g. via a degree in a related science) was not considered as an academic midwife in this study. This was because the further development of midwifery study programs and the academization of the midwifery profession in Germany is established from the primary qualification with an academic degree^11^. The authors are aware that this is a possible bias. Nevertheless, this distinction was deliberately used to explicitly focus on the primary academic midwifery qualification. The high dropout rate (307 questionnaires started, 193 completed) could be due to several reasons. In general, an increased refusal and dropout rate is to be expected in online surveys^32^. Another reason could be the extended length of the questionnaire. Also, lack of motivation or not understanding the questions could lead to dropout. Both the gender distribution (almost exclusively female participants) and the education of midwives (predominantly midwives educated in vocational school) resulted in a selection bias. However, it must be considered that the gender distribution of midwives in Germany is mainly female^33^. Also, the academization of the midwifery profession in Germany is still in the early stages of development^1^, which is why primarily non-academic midwives participated. A different timing of the survey, when academization is further advanced, is considered reasonable. Moreover, the study design was a limitation, as there was only one measurement point. However, the performed t-tests must be considered critically. The prerequisite of homogeneity of variance was not fulfilled in the significance test for students and trainee midwives. Furthermore, it was recommended that the samples have approximately the same size and are not too small (N1=N2 ≥30)^34^. Both t-tests involved very different sized groups [t-test for students (n=55) and trainees (n=7), and t-test for midwives educated at university (n=18) and vocational school (n=90)]. In conclusion, the non-significant results of the t-test did not generally mean that no differences existed between the two groups of academic and non-academic midwives.

### Implications for research and practice

The central finding of this study was that academic midwifery competencies were relevant for both academic and non-academic midwives, which was reflected in the single factorial solution. Moreover, there were no significant differences in the assessed relevance of academic midwifery competencies within these two groups. These findings support the relevance of academization of the midwifery profession for all midwives. Furthermore, the questionnaire could reliably and validly capture the assessed relevance of the academic competence of midwives. Based on the present publication, recommendations for future research as well as for practice could be outlined. Firstly, the prerequisites for Cronbach’s α were not completely fulfilled, which is why the reliability should be tested again with another reliability coefficient (e.g. Bollen’s ω or McDonald’s ω). Moreover, the survey instrument should be fundamentally modified. The item difficulty was not sufficient, which limits the quality of the items. Therefore, the items should be modified regarding their difficulty. In addition, the assessment instrument did not fully represent the construct. The single factor solution caused a high level of information loss. This was reflected in the elimination of items that were relevant in terms of their content. Thirdly, the t-tests should be repeated with a larger sub-sample as well as groups of equal size. Finally, a recommendation can be made regarding increased education and the inclusion of non-academic midwives in the academization of the midwifery profession. It is important to communicate the relevance of the skills taught in academic midwifery education, especially in terms of the need for evidence-based practice and the associated research skills of midwives. This is important because non-academic midwives will be midwifery educators in the academic context for several years^2^.

## CONCLUSIONS

This work represented a fundamental step in the future design of the academization of German midwifery education. The central result of the work was that there were no significant differences in the assessed relevance of academic midwifery competencies between the two groups. Both the academic and non-academic midwives considered academic midwifery competencies to be meaningful, which was shown within the one-factor solution. The assessment has been psychometrically tested and is able to measure the assessed relevance of the academic competence of midwives in a reliable way, so that further use of the questionnaire (in a modified form) can be recommended. The findings also suggest that there is a need for educating and engaging non-academic midwives about the aims and process of academization within their profession. On the one hand, this was crucial because already licensed, practicing midwives with non-academic training needed research skills and should be able to engage with evidence to achieve quality health care^31^. On the other hand, non-academic midwives will be educators in the academic context for several years^2^. Therefore, the involvement of non-academic midwives in the process of academization in Germany is essential. There is also a need for further high-quality research, involving both lay and other professional groups in competency research for academic midwives. Additionally, the project of the University of Tübingen ‘Good Midwife’ will be continued. In the future, it will be relevant to consider these data in the context of the expectations of pregnant women in order to obtain a comprehensive picture of the expectations of midwives’ competences.

## Data Availability

The data supporting this research are available from the authors on reasonable request.
