# Supplementary material for: Assessment of the relevance of midwifery competencies in academic education in Germany from the midwives’ perspective: A structural analysis of cross-sectional survey data
Source: Eur J Midwifery. 2023 Sep 1;7:22. doi: 10.18332/ejm/169658 (PMC10472291; doi:10.18332/ejm/169658)
Supplement: Supplementary file 1 [file EJM-7-22-s1.pdf]

**II KOMPETENZ 1 Entscheidungs-, Steuerungs- und Handlungskompetenz**	**höchste Priorität**	**hohe Priorität**	**eher hohe Prio-rität**	**eher niedrige Priorität**	**niedrige Priorität**	**keine Priorität**
II 1. Sie bleibt entscheidungs- und handlungsfähig in schnellwechselnden und komplexen Situationen und Notfällen unter Berücksichtigung individueller, situativer und institutioneller Faktoren.	1	2	3	4	5	6
II 2. Sie nimmt ihre Rolle als Primärversorgende im Rahmen der physiologischen Geburtshilfe, sowie ihre Zuständigkeit als Unterstützende des physiologischen Prozesses von Schwangerschaft, Geburt, Wochenbett und Stillzeit wahr.	1	2	3	4	5	6
II 3. Sie richtet die Betreuung an dem Ziel aus, die Gesundheit von Frau, Kind und Familie aufrechtzuerhalten, zu fördern, bzw. wieder herzustellen.	1	2	3	4	5	6
II 4. Sie gewährleistet bzw. unterstützt Prinzipien und Strukturen, die nachweislich einen positiven Einfluss auf die Sicherheit, Effektivität und Effizienz der Versorgung nehmen (z.B. Ruhe, Vertrauen, Intimität, Kontinuität).	1	2	3	4	5	6
II 5. Sie identifiziert veränderten Betreuungsbedarf in komplexen psychischen und sozialen Situationen, gewährleistet die akute Versorgung in Krisensituationen, sowie das Einleiten entsprechender langfristiger Maßnahmen.	1	2	3	4	5	6
II 6. Sie identifiziert und vermeidet Einflussfaktoren und Maßnahmen, die nachweislich den natürlichen Geburtsprozess beeinträchtigen und das Aufkommen von Pathologie begünstigen, d.h. die Hebamme unterlässt vermeidbare Interventionen, soweit dies im Rahmen der evidenzbasierten Medizin begründet werden kann, sodass Prozesse in einem physiologischen Rahmen gehalten werden können.	1	2	3	4	5	6
**III KOMPETENZ 2 Reflexionsfähigkeit**	höchste Priorität	hohe Priorität	eher hohe Prio-rität	eher niedrige Priorität	niedrige Priorität	keine Priorität
III 1. Sie reflektiert, vertieft und erweitert kontinuierlich ihr theoretisches und wissenschaftliches Fachwissen und ist in der Lage dies fallbezogen anzuwenden.	1	2	3	4	5	6
III 2. Sie ermittelt, plant, handelt, reflektiert und evaluiert im Sinne sicherer, effektiver und effizienter Versorgung in Abstimmung mit der zu betreuenden Person.	1	2	3	4	5	6
III 3. Sie identifiziert und berücksichtigt soziale, kulturelle, ökonomische, psychologische und	1	2	3	4	5	6

geschlechtsspezifische Einflussfaktoren auf die Frauen-, und Kindergesundheit.						
III 4. Sie entwickelt ein breites und vertieftes Verständnis rechtlicher Bedingungen und ethischer Prinzipien der Berufsausübung.	1	2	3	4	5	6
III 5. Sie entwickelt und reflektiert das Berufsverständnis und die daraus resultierenden Anforderungen vor dem Hintergrund persönlicher Bedürfnisse (Burnout Prophylaxe).	1	2	3	4	5	6
**IV KOMPETENZ 3**<br>**Analytisch-diagnostische Begründungsfähigkeit**	höchste Priorität	hohe Priorität	eher hohe Prio-rität	eher niedrige Priorität	niedrige Priorität	keine Priorität
IV 1. Sie erfüllt rechtliche Vorgaben und unterstützt die Entwicklung evidenzbasierter Standards.	1	2	3	4	5	6
IV 2. Sie begleitet und beobachtet den Verlauf fortwährend, identifiziert Situationen, die eine Unterstützung des physiologischen Prozesses erfordern, begründet klinische Entscheidungen und geburtshilfliches Handeln evidenzbasiert und führt geburtshilfliche Interventionen im Einverständnis der zu betreuenden Personen rechtzeitig und angemessen durch, um den physiologischen Verlauf wieder herzustellen.	1	2	3	4	5	6
IV 3. Sie schätzt geburtshilfliche Risiken fachlich richtig und rechtzeitig ein, trifft begründete Entscheidungen, kommuniziert diese den betroffenen Personen gegenüber und reagiert auf jede Abweichung, die eine Konsultation bzw. eine Überweisung zu einer anderen Hebamme, einem Arzt / Ärztin oder anderen Gesundheitsfachberufen erfordert.	1	2	3	4	5	6
IV 4. Sie nutzt intuitive Wahrnehmung und Erfahrung, um Individuen und Situationen rasch einzuschätzen und Entwicklungen abzusehen (interne Evidenz).	1	2	3	4	5	6
**V KOMPETENZ 4**<br>**Fachkompetenz – Geburtshilfliches Wissen**	höchste Priorität	hohe Priorität	eher hohe Prio-rität	eher niedrige Priorität	niedrige Priorität	keine Priorität
V 1. Die Hebamme verfügt über eine vertiefte Forschungskompetenz, d.h. sie ist in der Lage Studien zu verstehen und diese auch selbstständig durchzuführen.	1	2	3	4	5	6
V 2. Sie ist in der Lage Informationen systematisch zu erfassen, zu bewerten, zu interpretieren und daraus wissenschaftlich fundierte Urteile, unter Berücksichtigung gesellschaftlicher, wissenschaftlicher und ethischer Erkenntnisse, abzuleiten.	1	2	3	4	5	6

V 3. Die Hebamme verfügt über ausreichend Wissen über die Frauengesundheit, d.h. sie berücksichtigt individual- und bevölkerungsmedizinische Aspekte im Betreuungsalltag.	1	2	3	4	5	6
V 4. Sie überliefert traditionelles und erfahrungsbasiertes Hebammenwissen, ist in der Lage diesen Erkenntniszugang zu reflektieren und unterstützt die forschungsbasierte Überprüfung dessen.	1	2	3	4	5	6
V 5. Sie identifiziert berufsspezifische Probleme und Anforderungen und nutzt Lernnetzwerke und Methoden des Teamlernens (z.B. Supervision, Fallbesprechungen) um Problemlösungen und Argumente in ihrem Fachgebiet zu erarbeiten und weiterzuentwickeln.	1	2	3	4	5	6
V 6. Sie bildet sich eine fachlich fundierte Meinung und ist in der Lage diese auch in hierarchischen Strukturen argumentativ zu vertreten.	1	2	3	4	5	6
V 7. Sie identifiziert Anforderungen und Tätigkeitsbereiche, die einer Zusatzqualifikation und Spezialisierung bedürfen, um die berufliche Leistung auf einem hohen qualitativen Niveau zu sichern.	1	2	3	4	5	6
**VI KOMPETENZ 5 Methodenkompetenz**	höchste Priorität	hohe Priorität	eher hohe Prio-rität	eher niedrige Priorität	niedrige Priorität	keine Priorität
VI 1. Sie prüft, erweitert und vertieft kontinuierlich das geburtshilfliche Wissen und Verstehen auf Grundlage aktueller Fachliteratur und ist in der Lage relevante Wissensbestände auf dem Stand der Forschung zu erschließen und in das Wissen zu integrieren (externe Evidenz).	1	2	3	4	5	6
VI 2. Sie sammelt systematisch Informationen, analysiert jede Situation gezielt, führt geburtshilflich diagnostisch relevante Untersuchungen im Einverständnis der zu betreuenden Person durch bzw. veranlasst diese, stellt Diagnosen und prognostiziert den Verlauf.	1	2	3	4	5	6
VI 3. Sie setzt die sinnliche Wahrnehmung sowie manuelle Fertigkeiten gezielt ein und nutzt diese für anerkannte Diagnose-, und Behandlungstechniken (taktil-kinästhetisch, körpertherapeutisch) bzw. zu deren Weiterentwicklung.	1	2	3	4	5	6
VI 4. Sie gestaltet selbständig weiterführende Lernprozesse, und ist in der Lage neue Erkenntnisse in das berufliche Handeln zu integrieren.	1	2	3	4	5	6
VI 5. Die Hebamme verfügt über digitale Kompetenzen in den Bereichen Datenverarbeitung, Kommunikation,	1	2	3	4	5	6

Erstellung von Inhalten sowie Sicherheit und kann diese in allen Bereichen der Hebammentätigkeit anwenden.						
**VII KOMPETENZ 6 Beziehungsfähigkeit und Kommunikationskompetenz**	höchste Priorität	hohe Priorität	eher hohe Prio-rität	eher niedrige Priorität	niedrige Priorität	keine Priorität
VII 1. Sie strebt ein von Würde und Respekt gekennzeichnetes Vertrauensverhältnis an und ist in der Lage dies zu wahren (auch in komplexen und durch Anonymität gekennzeichneten Situationen).	1	2	3	4	5	6
VII 2. Sie erkennt und respektiert die individuellen emotionalen, sozialen, kulturellen und religiösen Bedürfnisse der Frau.	1	2	3	4	5	6
VII 3. Sie kommuniziert und interagiert auf Grundlage des professionellen Beziehungsaufbaus.	1	2	3	4	5	6
VII 4. Sie setzt sich bewusst mit asymmetrischen Beziehungen und Machtverhältnissen auseinander und reflektiert eigene Werte und Vorstellungen, sowie deren Einfluss auf das berufliche Handeln kritisch.	1	2	3	4	5	6
VII 5. Sie plant und evaluiert die Versorgung auf Grundlage einer professionellen Rollenbeziehung mit den Klienten/ Klientinnen und stellt optionale Handlungsvarianten zur Verfügung.	1	2	3	4	5	6
VII 6. Sie identifiziert den Bedarf der Frau an Unterstützung und verfügt über ein entsprechendes Rollenrepertoire um diesem im Rahmen des „therapeutischen Arbeitsbündnisses“ zu entsprechen.	1	2	3	4	5	6
VII 7. Sie respektiert die Eigenkompetenz und Autonomie der Frau und ihrer Familie und fördert deren Ressourcen Bewältigungsstrategien zu entwickeln (Empowerment).	1	2	3	4	5	6
VII 8. Sie ermöglicht und unterstützt die positive Bewältigung von Schwangerschaft, Geburt, Wochenbett und Stillzeit als wichtiges Lebensereignis von Frau, Kind und Familie.	1	2	3	4	5	6
VII 9. Sie erkennt Mutter und Kind als Einheit an und sichert die Gesundheitsversorgung des Kindes unter Einbezug der Mutter und der Familie.	1	2	3	4	5	6
VII 10. Sie ermöglicht und unterstützt den ressourcenorientierten Einbezug des familiären Umfeldes, so wie es von der Frau definiert wird.	1	2	3	4	5	6
VII 11. Sie identifiziert den Bedarf einer Frauen- und Familienorientierten Versorgung und stellt diesen in den Mittelpunkt professioneller Versorgungskonzepte.	1	2	3	4	5	6

VII 12. Sie respektiert das Recht der Frau und der Familie auf Aufklärung und Beratung und fördert die informierte Entscheidung.	1	2	3	4	5	6
VII 13. Sie informiert und klärt Jugendliche und Erwachsene bezüglich Sexualität und Elternschaft auf.	1	2	3	4	5	6
VII 14. Sie ist sensibilisiert für Anzeichen von Gewalt in der Familie, sexuellen Missbrauch oder Drogenkonsum bei der Frau und realisiert die Versorgung durch Vernetzung und Überweisung.	1	2	3	4	5	6
VII 15. Sie erkennt Versorgungsdefizite des Kindes in der Familie, ist sensibilisiert für Anzeichen von Missbrauch des Kindes und leitet entsprechende Maßnahmen zur Sicherung des Kindswohls ein.	1	2	3	4	5	6
**VIII KOMPETENZ 7<br>Intra- und interdisziplinäre Kooperation und Zuständigkeit**	höchste Priorität	hohe Priorität	eher hohe Prio-rität	eher niedrige Priorität	niedrige Priorität	keine Priorität
VIII 1. Sie entscheidet im Rahmen ihrer rechtlichen Zuständigkeit und individuellen Kompetenz, wann sie selbstverantwortlich handelt bzw. wann sie andere Fachpersonen hinzuzieht.	1	2	3	4	5	6
VIII 2. Sie zieht beim Auftreten pathologischer Befunde Arzte und Ärztinnen rechtzeitig hinzu und führt bis zu deren Eintreffen adäquate Notfallmaßnahmen aus.	1	2	3	4	5	6
VIII 3. Sie sichert in fachlich begründeten Fällen die Versorgung von Frauen und Kinder mit komplexen Anforderungen im Sinne der integrierten Versorgung.	1	2	3	4	5	6
VIII 4. Sie wird vielfältigen Anforderungen gleichzeitig gerecht, kann Prioritäten setzen und falls erforderlich Unterstützung einfordern bzw. Grenzen der Leistbarkeit realisieren.	1	2	3	4	5	6
VIII 5. Sie übernimmt ihre Verantwortung im geburtshilflichen Team und führt Anweisungen weisungsbefugter Personen (Arzte und Ärztinnen) zeitgerecht und effektiv durch.	1	2	3	4	5	6
VIII 6. Sie fördert und gestaltet eine optimale Zusammenarbeit sowohl innerhalb ihres Fachbereichs als auch interdisziplinär.	1	2	3	4	5	6
VIII 7. Sie dokumentiert patientenbezogene Daten sowie Planung, Durchführung und Evaluation des Betreuungsprozesses fachlich richtig und nachvollziehbar und ist in der Lage Befunde, Diagnosen und Maßnahmen sprachlich adäquat an Laien und Fachvertretern zu vermitteln.	1	2	3	4	5	6

**IX KOMPETENZ 8**<br>**Entwicklung und Organisation**	höchste Priorität	hohe Priorität	eher hohe Prio-rität	eher niedrige Priorität	niedrige Priorität	keine Priorität
IX 1. Sie definiert ihr Verständnis von Geburt und Geburtshilfe unter kritischer Berücksichtigung beruflich relevanter Theorien, Prinzipien und Methoden.	1	2	3	4	5	6
IX 2. Sie setzt sich für eine nationale und internationale Sozial- bzw. Gesundheitspolitik ein, die eine professionelle Versorgung von schwangeren Frauen, Gebärenden und Wöchnerinnen und ihren Kindern sichert.	1	2	3	4	5	6
IX 3. Sie bezieht sich auf ein definiertes berufliches Profil unter Berücksichtigung nationaler und internationaler Standards und Ethikkodexe.	1	2	3	4	5	6
IX 4. Sie identifiziert ethische Dilemmata und beteiligt sich an ethischen Entscheidungsfindungsprozessen auf gesellschaftlicher und individueller Ebene.	1	2	3	4	5	6
IX 5. Sie nimmt die professionelle Verantwortung und die persönliche Haftung gegenüber der Frau, dem Arbeitgeber, der Profession und der Gesellschaft wahr.	1	2	3	4	5	6
IX 6. Sie identifiziert und realisiert individuelle und institutionelle Arbeitsbedingungen, die eine sichere, effektive und effiziente Versorgung gewährleisten.	1	2	3	4	5	6
IX 7. Sie fördert das Ansehen des Berufsstandes und unterstützt berufspolitische Strategien.	1	2	3	4	5	6
IX 8. Sie identifiziert und analysiert die gesellschaftliche Relevanz der beruflichen Leistung und setzt sich für optimale Bedingungen der Berufsausbildung und Berufsausübung ein.	1	2	3	4	5	6
IX 9. Sie bietet Begleitung und Anleitung insbesondere für Auszubildende und Berufsanfänger und Berufsanfängerinnen.	1	2	3	4	5	6
IX 10. Sie organisiert die eigene Praxis nach rechtlichen, wirtschaftlichen und ökologischen Grundsätzen.	1	2	3	4	5	6
IX 11. Sie nutzt den Einsatz von Qualitätsmanagement (interne und externe Evaluation), um die Qualität der Leistung zu sichern.	1	2	3	4	5	6
IX 12. Sie unterstützt gesundheitsfördernde und präventive Programme und deren Entwicklung.	1	2	3	4	5	6
IX 13. Sie begründet ihre Zuständigkeit der autonomen und selbstverantwortlichen Primärversorgung im Rahmen von Familienplanung, Schwangerschaft, Geburt, Wochenbett und Stillzeit auf rechtlicher Zuständigkeit, Qualifizierung und Kompetenz, dem Versorgungsbedarf	1	2	3	4	5	6

der Frau, Evidenzen mit dem Nachweis des optimalen „outcomes“ für Frauen, Kinder und Familien.	

© 2023 Kranz A. Et al.
